# Adverse childhood experiences and incident coronary heart disease: a counterfactual analysis in the Whitehall II prospective cohort study

**DOI:** 10.1016/j.ajpc.2021.100220

**Published:** 2021-06-24

**Authors:** Mifuyu Akasaki, Owen Nicholas, Jessica Abell, Carlos A. Valencia-Hernández, Rebecca Hardy, Andrew Steptoe

**Affiliations:** a Social Research Institute, Institute of Education, University College London, London, UK; b Department of Behavioural Science and Health, Institute of Epidemiology and Health Care, University College London, London, UK; c Department of Statistical Science, Faculty of Mathematical and Physical Sciences, University College London, London, UK; d Department of Epidemiology and Public Health, Institute of Epidemiology and Health Care, University College London, London, UK

**Keywords:** Adverse childhood experiences, Incident coronary heart disease, Counterfactual analysis, Prospective longitudinal study

## Abstract

**Objectives:**

Adverse childhood experience is thought to be associated with risk of coronary heart disease, but it is not clear which experiences are cardiotoxic, and whether risk increases with the accumulation of adverse childhood experiences.

**Methods:**

Participants were 5149 adults (72.6% men) in the Whitehall II cohort study. Parental death was recorded at phase 1 (median age in years 44.3), and 13 other adverse childhood experiences at phase 5 (55.3). We applied Cox proportional hazards regression with person-time from phase 5 to examine associations of adverse childhood experiences with incident coronary heart disease. We predicted hazard ratios according to count of the experiences, and examined dose-response effect. We finally estimated reduction of coronary heart disease in a hypothetical scenario, the absence of adverse childhood experiences.

**Results:**

Among study participants, 62.9% had at least one adversity, with “financial problems” having the highest prevalence (26.1%). There were 509 first episodes of coronary heart disease during an average 12.9 years follow-up. Among 14 adverse childhood experiences in a multiply adjusted model, “parental unemployment” showed the highest hazard of coronary heart disease incidence (hazard ratio; 95% confidence interval: 1.53; 1.16 to 2.02). No dose-response effect was observed (constant for proportionality in hazard ratio: 1.05, 0.99 to 1.11). Based on the estimates of final model, in the absence of childhood adversities, we estimated a 6.0% reduction in coronary heart disease (0.94; 0.87 to 1.01), but the confidence interval includes one.

**Conclusion:**

Although individual adverse childhood experiences show some association with coronary heart disease, there is no clear relationship with the number of adverse experiences. Further research is required to quantify effects of multiple and combinations of adverse childhood experiences considering timing, duration, and severity.

## Introduction

Coronary heart disease is the leading cause of death and disability worldwide [1]. A large body of research has identified a series of risk factors for the development and progression of coronary heart disease. These studies usually focus on risk factors in adulthood, yet coronary heart disease represents a long-term disease process, from the development of atherosclerosis, subclinical disease, to its clinical manifestations. This natural history of coronary heart disease appears to start early in life [2,3]. One major finding is that more disadvantaged socioeconomic position in childhood is related to increased risk of coronary heart disease in adulthood [4]. This association may be explained in part by those who grow up in disadvantaged circumstances being more likely to engage in health risk behaviours than those who do not [5]. However, findings from physiological studies during the past decades suggest an additional possible underlying mechanism, namely the biological embedding of psychologically stressful events in childhood [6,7], which are more likely to occur among those in a more disadvantaged circumstance [8].

Adverse childhood experiences (ACEs), traumatic and stressful events in childhood and adolescence, appear to be associated with unfavourable brain development and function, resulting in potential negative behavioural and physiological changes, and unfavourable stress reactivity over life [9], [10], [11]. A meta-analysis estimated that those who had ACEs were three times more likely to be smokers, six times as likely to drink alcohol problematically, as well as four times more likely to have depression [12]. Those who experienced ACEs have also be found to be more likely to have hypertension, obesity and hyperlipidaemia later in life [13]. Failures of adaptation to stress and insufficient recovery from the stress, in addition to engagement in health risk behaviours, may play a substantial role in the development and progression of coronary heart disease [9,14,15].

Findings for associations of ACEs with coronary heart disease are, however, mixed; some studies documented positive associations, most of which also reported dose-response associations [16], [17], [18], [19], [20], [21], [22], but not all [23], [24], [25], [26]. In population based longitudinal studies, in which participants were linked to electronic health records, there were no associations between ACEs and coronary heart disease [23,24]. On the other hand, an approximate doubling of risk of coronary heart disease was reported in a meta-analysis based on cross-sectional studies, in which ACEs were measured at the same time as assessment of coronary heart disease morbidity [12]. Differences in the measurement of ACEs make interpretation of findings across studies challenging [27]. Some studies included markers of childhood socioeconomic position as a type of ACE, although being in a disadvantaged socioeconomic position is not necessarily equivalent to adverse events. Moreover, the majority of existing studies have used a count of ACEs, as the exposure. This approach assumes that each experience has an equal effect on the outcome with no correlation between experiences [27], even though ACEs are likely to co-occur in an individual [12]. A major advantage of an ACE score is that it permits the examination of dose-response associations, as well as defining the total exposure to ACEs in a simple manner. On the other hand, an approach that recognises different effect sizes in a simultaneously adjusted model [28,29] tackles some of the challenges of ACE scores [27,30].

To date, there are few longitudinal studies with follow-up from the time when ACEs are assessed, linked with central registry records of coronary heart disease. It also remains unclear whether specific ACEs are particularly important, or whether there is a dose-response association between ACEs and coronary heart disease after taking different effect sizes into account. Additionally, given that any such association may be causal, and that some ACEs are modifiable, it is important to quantify to what extent the elimination of ACEs can be beneficial for coronary heart disease prevention [31], which no longitudinal studies have yet examined.

Accordingly, the objectives of this study are (i) to examine association of each type of ACEs with incident coronary heart disease in adulthood, and its dose-response association, and (ii) to quantify the reduction in risk of incident coronary heart disease in the absence of ACEs, using a counterfactual approach.

## Methods

### *Study population*

**Fig. 1:**
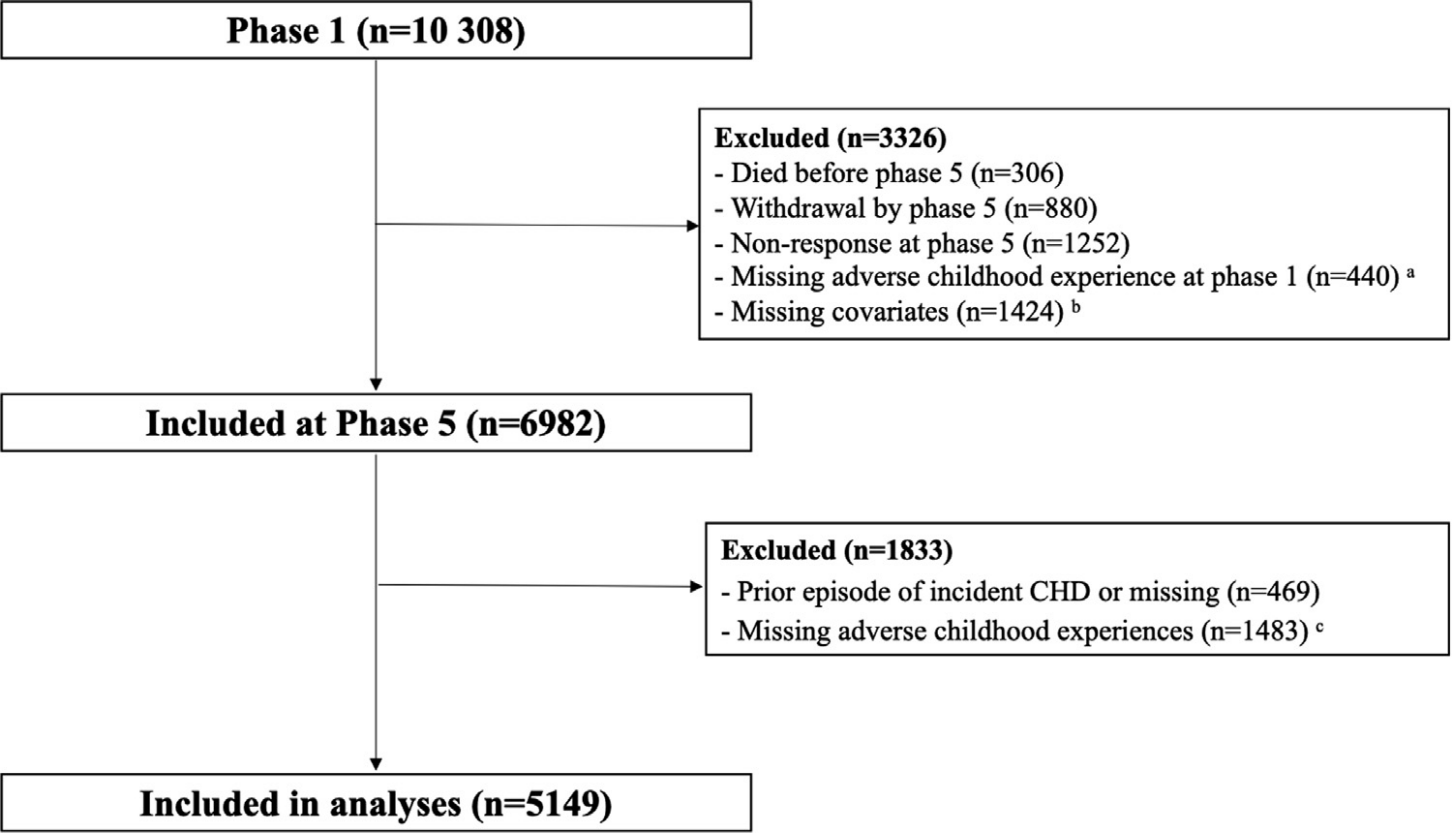
Flow chart of participants’ recruitment. ^a^Parental death, *n*=440 ^b^Sex, *n*=0; age, *n*=0; ethnicity, *n*=92; childhood SES, *n*=1410 ^c^Maternal separation 1yr+, *n*=694; hospitalisation 4wks+, *n*=836; divorce, *n*=842; mental illness and alcohol problems, n=848; argument, *n*=840; unemployment, n=851; financial problems, *n*=807; physical abuse, *n*=851; orphanage, *n*=863; lack of attachment with mothers, *n*=809; lack of attachment with fathers, *n*=1096; mother's harsh punishment, *n*=771; father's harsh punishment, *n*=1042.

The Whitehall II study is a prospective cohort study established in 1985 when 10 308 participants (men 6895; women 3413) aged 35 to 55 were recruited from 20 government departments in London. The Joint University College London/University College London Hospital Committees on the Ethics of Human Research has approved the Whitehall II study. To date, 12 phases of data collection featuring medical examination and questionnaire administration have been completed (mean inter-phase period 3 years) and the response rate was over 65% across all phases. We included 5149 participants who have no missing values in ACEs, confounders, and incident coronary heart disease (Fig. 1).

### *Assessment of Adverse Childhood Experiences*

We used data from phases 1 (1985-1988) and 5 (1997-1999) when adverse events in childhood before the age of 18 (between 1948 and 1968) were assessed retrospectively using a questionnaire. Among 20 items, five items were adopted from the European Prospective Investigation into Cancer and Nutrition study (EPIC); [32] two from the Childhood Experience of Care and Abuse (CECA) interview; [33] 10 items from the Midlife Development in the United States (MIDUS) study; [34] and the rest was designed for the Whitehall II study. Cronbach α estimates from the MIDUS items indicated internal consistency in two groups of items (four items per group), from which we created two summary score variables to take severity of these experiences into account. Consequently, 14 variables were derived. The original items with codes, phases of data collection, and details of derived variables are presented in a supplementary file 1 (table S1).

**ACEs:** 14 variables were derived which consisted of *binary ACEs (0: no; 1: yes)*; “maternal separation 1yr+”, “hospitalisation 4wks+”, “divorce”, “unemployment”, “mental illness and alcohol problems”, “physical abuse”, “arguments between parents”, “orphanage”, “financial problems”, “parental death”, and of *ordinal ACEs (range)*; “lack of attachment to mother” (4 to 16), “lack of attachment to father” (4 to 16), “mother's harsh punishment” (1 to 4), and “father's harsh punishment” (1 to 4).

### *Confounders*

We identified potential confounders from existing studies [22,29,35], and a direct acyclic diagram (supplementary file 1, figure S1). Sex, age in years, and ethnicity (white, non-white) were derived from phase 1. Missing values in ethnicity were replaced with responses from phase 5. Fathers’ occupational grade was used as a marker of childhood socioeconomic position, derived from phase 1, and missing values were replaced with responses at phase 6. Childhood socioeconomic position was categorised as professional, managerial/technical, skilled-non-manual, skilled-manual, partly skilled, and unskilled.

### *Ascertainment of incident Coronary Heart Disease*

Incident coronary heart disease was identified by a combination of data collected during the medical examination, and linkage of study participants to records from the National Health Service (Hospital Episode Statistics, HES) [36]. Data collected from the medical examination were based on a 12-lead resting electrocardiodiogram recording, and self-reported incident coronary heart disease which was confirmed by information by general practitioners or manual retrieval of hospital records. Non-fatal myocardial infarction, definite angina, coronary artery bypass grafting, and percutaneous transluminal coronary angiography were included in the ascertainment. The HES-ascertainment is based on the linkage with the records from hospitalisations for non-fatal coronary heart disease as a primary or secondary diagnosis, defined by the International Classification of Disease (ICD) 9 (codes 410-414) and 10 (codes I20 – I25), or procedures K40-K49, K50, K75, and U19. The dates of events were identified through the records used to confirm the events. Incident coronary heart disease in this study refers to the first episode as observed between phase 5 (1997-1999) to phase 11 (2012-2013).

### *Statistical analysis*

We described the prevalence of ACEs, incident coronary heart disease, and covariates among those excluded from the study, and the study sample (n=5149). We computed hazard ratios (HRs) and 95% Confidence Intervals (CIs) of the incident coronary heart disease in associations with ACEs by applying Cox proportional hazard regression. The time scale was person-time starting from the baseline in phase 5. In preliminary analyses, we included an interaction term for sex with each ACE to examine whether it modified the association of ACEs with the incident coronary heart disease. With no such evidence, pooled estimations are presented. We computed variance inflation factors for each ACE, all of which were well below acceptable threshold (1.02 to 1.57). We therefore included all type of ACEs in a model, adjusted for sex and age, and finally additionally adjusting for ethnicity and childhood socioeconomic position, assuming that all ACEs are contemporaneous. Based on the estimates in the final model, we predicted the average HRs and 95% CIs according to the count of ACEs with confounders held constant, and estimated a dose-response effect for the average HRs against the count of ACEs. To obtain the observed count of ACEs for each individual, we coded the worst quartile and worst Likert scale of ordinal ACEs as 1 (yes), otherwise 0 (no), while when estimating the average HRs and 95% CIs we coded the worst quartile as the difference from the highest score in the third quartile in order to account for severity. We finally estimated the reduction in hazard of coronary heart disease in the absence of ACEs as the counterfactual scenario versus the observed. In carrying out this estimation, all covariates were set to the means.

As sensitivity analyses, we estimated HRs and 95% CIs for each ACE in a model in which one adversity was included at a time with adjustment for the same confounders in the main analysis. We also fitted the model with ACEs score (count of ACEs) with the same adjustment.

We used Stata MP version 16.0 for all analyses, apart from estimating a dose-response for count of ACEs. Calculation methods for the dose-response effect is presented in a supplementary file 2.

## Results

The selection of the study sample is presented in Fig. 1. Of the 10 308 participants at phase 1, 7870 participants took part in phase 5. By excluding those who had missing values in ACEs assessed at phases 1 and 5, confounders, along with those with a prior episodes of incident coronary heart disease, the number in the analytical sample was 5149 (men: 72.6%).

In Table 1 we present the characteristics of the study sample according to each ACE. Compared with those excluded, the study sample was more likely to be male, be younger, be of White ethnicity, and be in a non-manual childhood socioeconomic position. The prevalence of ACEs was lower among those included in the study. Among the study sample, 62.9% had at least one ACE. The highest prevalence was observed for financial problems (26.1%), followed by arguments between parents (19.5%). In Table 2 we show distributions of covariates according to the count of ACEs. Women were more likely than men to have a larger count of ACEs. There was a higher proportion of those who had a father in a manual occupation in the larger count of ACEs.

**Table 1:** Characteristics of study population according to inclusion in the present analytical sample.

	**Excluded sample (*n*=5159)**^a^	**Study sample (*n*=5149)**

**Exposure**^b^		
No adverse childhood experiences, *n (%)*	-	1908 (37.1)
Maternal separation 1yr+, *n (%)*	427 (22.6)	520 (10.1)
Parental death, *n (%)*	852 (18.1)	370 (7.2)
Hospitalisation 4wks+, *n (%)*	285 (16.6)	627 (12.2)
Divorce, *n (%)*	198 (11.6)	99 (1.9)
Mental illness and alcohol problems, *n (%)*	116 (6.8)	304 (5.9)
Arguments between parents, *n (%)*	378 (22.1)	1003 (19.5)
Unemployment, *n (%)*	229 (13.5)	504 (9.8)
Financial problems, *n (%)*	655 (37.4)	1342 (26.1)
Physical abuse, *n (%)*	58 (3.4)	119 (2.3)
Orphanage, *n (%)*	69 (4.1)	28 (0.5)
Lack of attachment to mothers, *median (IQR)*	8 (6 to 10)	8 (6 to 10)
Lack of attachment to fathers, *median (IQR)*	10 (8 to 12)	10 (8 to 12)
Mother's harsh punishment, *median (IQR)*	2 (1 to 2)	2 (1 to 2)
Father's harsh punishment, *median (IQR)*	2 (1 to 3)	2 (1 to 3)
		
**Outcome**		
First episode of coronary heart disease from phase 5	271	509
		
**Covariates**		
Sex, *n (%)*		
Men	3158 (61.2)	3737 (72.6)
Women	2001 (38.8)	1412 (27.4)
Age in years at baseline, *median (IQR)*	45.2 (40.1 to 51.1)	43.6 (39.3 to 49.5)
Ethnicity, *n (%)*		
White	4342 (85.7)	4839 (94.0)
Non-white	725 (14.3)	310 (6.0)
Childhood socioeconomic position, *n (%)*		
Non-manual	2073 (55.3)	3109 (60.4)
Manual	1676 (44.7)	2040 (39.6)

a Proportion was calculated with the number of responders to each item as denominator, which differed across items. Due to the differences in the number of denominator across items, a proportion of “No adverse childhood experiences” is not available

b Adverse childhood experiences are not mutually exclusive

**Table 2:** Distribution of covariates according to the count of adverse childhood experiences (ACEs).

	**ACEs**^a^			
	**0**	**1**	**2**	**3+**
*n*	*1908*	*1262*	*895*	*1084*
Sex, *n (%)*				
Men	1490 (78.1)	925 (73.3)	630 (70.4)	692 (63.8)
Women	418 (21.9)	337 (26.7)	265 (29.6)	392 (36.2)
Age in years at baseline, *mean ± SD*	43.9 ± 5.9	44.5 ± 6.0	44.7 ± 6.1	44.9 ± 5.9
Ethnicity, *n (%)*				
White	1816 (95.2)	1185 (93.9)	833 (93.1)	1005 (92.7)
Non-white	92 (4.8)	77 (6.1)	62 (6.9)	79 (7.3)
Childhood socioeconomic position,^b^*n (%)*				
Non-manual	1308 (68.6)	763 (60.5)	518 (57.9)	520 (48.0)
Manual	600 (31.5)	499 (39.5)	377 (42.1)	564 (52.0)

^a^In this table, the count of ACEs were categorised into four groups for convenience to describe distribution of covariates

^b^Non-manual: professional, managerial/technical, skilled-non-manual; Manual: skilled-manual, partly skilled, and unskilled

**Fig. 2:**
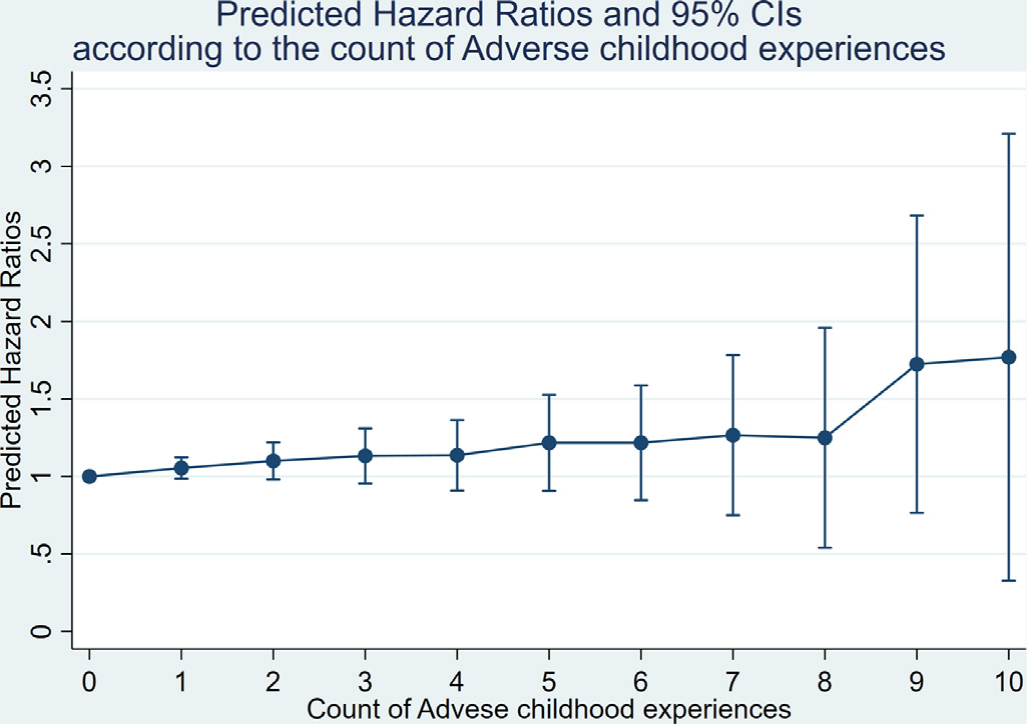
Predicted hazard ratios and 95% confidence intervals (CIs) of incident coronary heart disease by the counts of adverse childhood experiences (ACEs)^a.^ ^a^Adverse childhood experiences in the presented study range from zero to 10.

A mean duration of follow-up of 12.9 years (standard deviation 4.5) gave rise to 509 first episodes of coronary heart disease. In Table 3 we report the HRs and 95% CIs of coronary heart disease in the associations with ACEs. In the fully adjusted model those who experienced maternal separation had 1.33 times higher hazard of incident coronary heart disease (HR; 95% CI: 1.33; 1.03 to 1.73) than those who did not. Also, people whose parents were unemployed had 1.53 times higher hazard of incident coronary heart disease (1.53; 1.16 to 2.02) than those whose parents were employed. Predicted HRs from the fully adjusted model by the count of ACE are presented in Fig. 2. Our analysis showed that hazard of incident coronary heart disease would increase by 5.0% as each count of ACEs increases, but 95% CIs just included one (constant for proportionality in HR 1.05; 95% CI 0.99 to 1.11). We estimated a 6.0% (0.94; 0.87 to 1.01) reduction in hazard of coronary heart disease in the absence of all ACEs as a counterfactual scenario against the observed distribution of ACEs, however 95% CIs crossed one.

**Table 3:** Hazard ratios (HRs) and 95% confidence intervals (CIs) for the association of adverse childhood experiences (ACEs) with incident coronary heart disease (CHD) in a model including all ACEs simultaneously in the Whitehall II study (*n*=5149).

^a^ A model in which all ACEs were adjusted for simultaneously. “Lack of attachment to mothers/fathers” and “Mothers/fathers’ harsh punishment” are ordinal, and the other variables are binary in which reference groups are people who have no corresponding ACEs

^b^ Number of incident coronary heart disease among people in the worst quartile

^c^ Number of incident coronary heart disease among people who answered “great deal” in 4-likert scale

^d^ Risk ratios (RRs) for the associations of each type of ACEs with incident coronary heart disease

^e^ A model adjusted for sex, age, ethnicity, and childhood socioeconomic position

Results of sensitivity analyses are presented in a supplementary file 1 (table S2). Models in which one adversity was added at a time showed that, although there were slight differences in effects sizes from the main analysis in which other ACEs were adjusted, only maternal separation and parental unemployment had positive associations with incident coronary heart disease, consistent with the main analysis. There was no evidence that the ACEs score showed an association with incident coronary heart disease.

## Discussion

We found that only two types of ACEs were independently associated with incident coronary heart disease later in life. We found no dose-response effects between the counts of ACEs and incident coronary heart disease. We estimated a 6.0% reduction in coronary heart disease in the absence of ACEs, but 95% CIs for this protective association included one in the present study.

The percentage of people who experienced at least one ACE in our study was 62.9%, in close agreement with a systematic review of this field [37]. Our results indicate that only parental unemployment and maternal separation are independently associated with increased risk of coronary heart disease. It may be that specific ACEs have different mechanisms through which they influence coronary heart disease. Unemployment in adulthood has been shown to be related to increased risk of coronary heart disease possibly due to cumulative chronic stress [38], but our study shows unemployment may also have intergenerational effects. One of the potential pathways from parental unemployment to adult coronary heart disease may be mediated by children's educational attainment, and subsequent adult socioeconomic position [39], an established risk factor of coronary heart disease [4]. On the other hand, maternal separation may partly involve alterations in biological mechanism during a sensitive period of early life as shown in previous human [40] and animal studies [41].

To address challenges of ACEs score as described in introduction, we fitted a model including all ACEs simultaneously similar to some previous research [28,29], and then predicted hazard ratios according to the count of ACEs in a continuous scale [42], retaining the information of severity in ordinal ACEs. Given that ACEs are likely clustered and co-occurring, estimated risks for each ACE may provide limited information on their own [43,44], while estimates for the overall effect of ACEs may be more reliable due to their smaller standard errors. Our counterfactual estimate for the overall effect of all ACEs, 6.0% (HR 0.94; 95% CI: 0.87 to 1.01), is close to a finding from a systematic review based on cross-sectional studies that reported an approximately 10% reduction [37]. Despite this relatively small figure, particularly when comparing with corresponding reported figures for health risk behaviours, around 20 to 35% [37], 6.0% is noteworthy when the loss of disability-adjusted life years due to coronary heart disease is taken into consideration [1].

The mixed results from existing studies can be partly explained by the differences in the measurement of ACEs and coronary heart disease, definition of ACEs, conceptual pathways, and study design [27,45]. A questionnaire developed by the Center for Disease Control and Prevention has been used in some studies [17], [18], [19], but as in the Whitehall II study, sources of items vary across studies, providing non-comparable findings by capturing different aspects of ACEs. This variation may also be due to the ambiguity of ACEs definition, as seen in a debate about inclusion of childhood socioeconomic position as an ACE [27]. Some studies included adjustment for mental health and health behaviours, which can be mediators. We thus adjusted for variables considered to confound the association between ACEs and coronary heart disease, although we are aware of other potential confounders (e.g., birth weight), not available in the cohort study. Our findings are consistent with longitudinal studies using electronic health records and retrospectively measured ACEs reporting little evidence for the association [23,24]. It would be interesting to examine associations of prospectively measured ACEs with coronary heart disease, and to compare findings with longitudinal studies in which retrospectively measured ACEs were used.

This study is limited by the retrospectively measured ACEs, which may be influenced by the health status (e.g. depression) at the time of assessment. Despite possible discrepancies between prospective and retrospective measurement of ACEs [46], our findings can be interpreted in the context of retrospectively measured ACEs in adulthood, given that prospective measurement could also be biased (e.g., under-report of sexual abuse), Our study was limited to 14 ACEs, and did not include distal relationships (i.e., school peers), societal or environmental events, the duration, and age at the occurrence of ACEs. However, we were unable to investigate these because of a lack of information. We cannot rule out the possibility of over-adjustment because some ACEs potentially lie on the causal pathway (e.g., family financial problems and parental mental illness). Almost half of participants were excluded from the study because of missing values in covariates, having died before or not having responded at phase 5 when most of ACEs were assessed, or having had coronary heart disease already before phase 5. In our study, missingness due to those who had a prior episode of coronary heart disease, or subsequent deaths with missing ACEs information can bias the estimates [47].

## Conclusion

Almost two thirds of population have a legacy of ACEs throughout life. Our research demonstrates that the majority of ACEs may not have negative associations with the development of coronary heart disease later in life. In the present study, there was no strong evidence for dose-response effect in the association with count of ACEs, nor an overall effect of accumulation of ACEs on coronary heart disease. Further research is required to examine the role of multiple combinations of ACEs on coronary heart disease, with more focus on the development of coronary heart disease, from the aspects of timing, duration, and severity to represent dynamics of adverse experiences in childhood.

### Declarations

**Contributors:** MA conceived the initial idea for the work and elaborated this with all authors. MA, JA, and AS designed the study. MA, ON, and RH undertook statistical analysis. All authors contributed to the draft of the work, to the critical revision, and to the final approval of the version to be published. All authors had agreement to be accountable for the accuracy and integrity of the study. The corresponding author affirms that all authors meet authorship criteria recommended in the International Committee of Medical Journal Editors (ICMJE) Recommendations for the Conduct, Reporting, Editing, and Publication of Scholarly Work in Medical Journals 2013. The corresponding author attests that all listed authors meet authorship criteria and that no others meeting the criteria have been omitted.

**Funding:** The Whitehall II study was supported by the UK Medical Research Council (MRC, K013351; R024227), the US National Institute on Aging (NIA, R56AG056477; R01AG0564779), and the British Heart Foundation (32334). The funders did not contribute to the study design, data collection, analysis, interpretation, drafting, nor the decision to publish the study.

**Declaration of Competing interest:** None declared.

**Availability of data and material:** Data of the Whitehall II study are available to the scientific community. Data sharing policy is available at https://www.ucl.ac.uk/epidemiology-health-care/research/epidemiology-and-public-health/research/whitehall-ii/data-sharing.

**Code availability:** Available from the authors on request.

**Ethical approval:** It was not required since the study used secondary data. The Joint University College London/University College London Hospital Committees on the Ethics of Human Research has approved the Whitehall II study.

**Acknowledgments**. We thank all of the participating civil service departments and their welfare, personnel, and establishment officers; the British Occupational Health and Safety Agency; the British Council of Civil Service Unions; all participating civil servants in the Whitehall II study; and all members of the Whitehall II study team. The Whitehall II Study team comprises research scientists, statisticians, study coordinators, nurses, data managers, administrative assistants and data entry staff, who make the study possible.

**Transparency statement:** MA affirms that the manuscript is an honest, accurate, and transparent account of the study being reported; that no important aspects of the study have been omitted; and that any discrepancies from the study as originally planned (and, if relevant, registered) have been explained.

## Appendix Supplementary materials

Image, application 1

Image, application 1
