# Supplementary material for: Adverse childhood experiences and incident coronary heart disease: a counterfactual analysis in the Whitehall II prospective cohort study
Source: Am J Prev Cardiol. 2021 Jun 24;7:100220. doi: 10.1016/j.ajpc.2021.100220 (PMC8387301; doi:10.1016/j.ajpc.2021.100220)
Supplement: Supplementary file 1 [file mmc1.docx]

**Table S1. Questionnaires of adverse childhood experiences up to age 16**

| **Questions** | **Phase** | **Origins of items**^a^ | **Original code** | **Derived code** |
| --- | --- | --- | --- | --- |
| Were you ever separated from your mother for a year or more as a child | 5 | HLEQ | 0 (No), 1 (Yes) | 0 (No), 1 (Yes) |
| You spent 4 or more weeks in hospital | 5 | HLEQ | 0 (No), 1 (Yes) | 0 (No), 1 (Yes) |
| Your parents were divorced | 5 | HLEQ | 0 (No), 1 (Yes) | 0 (No), 1 (Yes) |
| Your father/mother were unemployed when they wanted to be working | 5 | HLEQ | 0 (No), 1 (Yes) | 0 (No), 1 (Yes) |
| Your parent(s) were mentally ill or drank so often that it caused family problems | 5 | HLEQ | 0 (No), 1 (Yes) | 0 (No), 1 (Yes) |
| You were physically abused by someone close to you | 5 | HLEQ | 0 (No), 1 (Yes) | 0 (No), 1 (Yes) |
| Your parents very often argued or fought | 5 | CECA | 0 (No), 1 (Yes) | 0 (No), 1 (Yes) |
| You were in an orphanage/children’s home | 5 | CECA | 0 (No), 1 (Yes) | 0 (No), 1 (Yes) |
| Your family had continuing financial problems | 5 | Whitehall II | 0 (No), 1 (Yes) | 0 (No), 1 (Yes) |
| Is your natural father/mother still alive? How old were you when he/she died? | 1 | Whitehall II | 0 (No), 1 (Yes) Age as continuous | 0 (No),  1 (if either or both parents died up to age 16) |
| How much did she understand your problems and worries? | 5 | MIDUS | 1 (great deal); 2 (quite a lot); 3 (a little); 4 (not at all) | Summary score; range 4 to 16 ^b^ Standardised Cronbach’α: 0.88 |
| How much could you confide in her about things that were bothering you? | 5 |  | 1 (great deal); 2 (quite a lot); 3 (a little); 4 (not at all) |  |
| How much love and affection did she give you? | 5 |  | 1 (great deal); 2 (quite a lot); 3 (a little); 4 (not at all) |  |
| How much time and attention did she give you when you needed it? | 5 |  | 1 (great deal); 2 (quite a lot); 3 (a little); 4 (not at all) |  |
| How much did he understand your problems and worries? | 5 |  | 1 (great deal); 2 (quite a lot); 3 (a little); 4 (not at all) | Summary score; range 4 to 16 ^b^ Standardised Cronbach’α: 0.88 |
| How much could you confide in him about things that were bothering you? | 5 |  | 1 (great deal); 2 (quite a lot); 3 (a little); 4 (not at all) |  |
| How much love and affection did he give you? | 5 |  | 1 (great deal); 2 (quite a lot); 3 (a little); 4 (not at all) |  |
| How much time and attention did he give you when you needed it? | 5 |  | 1 (great deal); 2 (quite a lot); 3 (a little); 4 (not at all) |  |
| How harsh was she when she punished you? | 5 |  | 1 (great deal); 2 (quite a lot); 3 (a little); 4 (not at all) | 1 (not at all); 2 (a little); 3 (quite a lot); 4 (great deal) ^b^ |
| How harsh was he when she punished you? | 5 |  | 1 (great deal); 2 (quite a lot); 3 (a little); 4 (not at all) | 1 (not at all); 2 (a little); 3 (quite a lot); 4 (great deal) ^b^ |

^a^ HLEQ: Health and Life Experiences Questionnaire^1^, developed for the European Prospective Investigation into Cancer and Nutrition (EPIC); CECA: Childhood Experience of Care and Abuse interview^2^; MIDUS: Midlife Development in the United States study.^3^

^b^ In computation of summary ACEs score and population attributable fractions, the worst quartile was coded as 1 (Yes), otherwise coded as 0 (No).

**Table S2. Hazard ratios (HRs) and 95% confidence intervals (CIs) in a separate model with one adversity at a time, and in a model with a cumulative ACEs score, in the association with incident coronary heart disease (CHD)**

|  | **No. CHD** | **HR**^a^ | **95% CI**^a^ |
| --- | --- | --- | --- |
| **A model with one adversity at a time** |  |  |  |
| Maternal separation 1yr+ | 76 | 1.31 | (1.02, 1.68) |
| Parental death | 40 | 0.96 | (0.69, 1.33) |
| Hospitalisation 4wks+ | 75 | 1.22 | (0.95, 1.56) |
| Divorce | 11 | 1.17 | (0.64, 2.13) |
| Mental illness and drunk | 28 | 1.01 | (0.69, 1.47) |
| Arguments between parents | 93 | 0.99 | (0.79, 1.24) |
| Unemployment | 71 | 1.44 | (1.11, 1.85) |
| Financial problems | 138 | 1.02 | (0.84, 1.25) |
| Physical abuse | 10 | 0.88 | (0.47, 1.64) |
| Orphanage | 4 | 1.40 | (0.52, 3.76) |
| Lack of attachment with mothers | 96^b^ | 1.01 | (0.98, 1.04) |
| Lack of attachment with fathers | 86^b^ | 0.99 | (0.96, 1.02) |
| Mother’s harsh punishment | 13^c^ | 0.99 | (0.88, 1.11) |
| Father’s harsh punishment | 37^c^ | 1.10 | (0.99, 1.21) |
|  |  |  |  |
| **A model with a cumulative ACEs score** |  |  |  |
| ACEs score | 509 | 1.04 | (0.98, 1.10) |

^a^ Adjusted for sex, age, ethnicity, and childhood socioeconomic position

^b^ Number of incident CHD among those who answered “Not at all,” but the model was fit with original variables in 4-likert scale

^c^ Number of incident CHD among those in the worst quartile, but the model was fit with original variables ranging from 1 to 4

**Figure S1. Directed acyclic diagram for the association between adverse childhood experiences and incident coronary heart disease**

Adverse childhood experiences

Incident coronary

heart disease

Sex

Age

Ethnicity

Childhood socioeconomic position

**Reference**

1. Wainwright NW, Surtees PG. Childhood adversity, gender and depression over the life-course. *Journal of affective disorders.* 2002;72(1):33-44.

2. Bifulco A, Brown GW, Harris TO. Childhood Experience of Care and Abuse (CECA): a retrospective interview measure. *Journal of child psychology and psychiatry, and allied disciplines.* 1994;35(8):1419-1435.

3. Shaw BA, Krause N, Chatters LM, Connell CM, Ingersoll-Dayton B. Emotional support from parents early in life, aging, and health. *Psychology and aging.* 2004;19(1):4.
