# Supplementary material for: Adverse childhood experiences and incident coronary heart disease: a counterfactual analysis in the Whitehall II prospective cohort study
Source: Am J Prev Cardiol. 2021 Jun 24;7:100220. doi: 10.1016/j.ajpc.2021.100220 (PMC8387301; doi:10.1016/j.ajpc.2021.100220)
Supplement: Supplementary file 2 [file mmc2.docx]

**Marginal log-hazard for counterfactual ACEs intervention**

Let the ith participants covariate values be denoted ${(x)}_{i}$, and let their counterfactual covariate values be denoted ${(x')}_{i}$,

For the population, the marginal log-hazard for the counterfactual ACEs intervention is

$$\frac{1}{N}\sum_{i} \beta^{T}(\left( x' \right)_{i}- {(x)}_{i})= \beta^{T}\frac{1}{N}\sum_{i} (\left( x' \right)_{i}- {(x)}_{i})$$

where the sum is over the N participants. Note that in the sum, for any non-ACE variable $x_{j}$, $\left( x_{j}' \right)_{i}- \left( x_{j} \right)_{i}=0$, so the only variables which contribute to the margin are the ACE variables, and the only coefficients the corresponding ACE coefficients.

Thus, we set all the non-ACE variables equal to zero, before running the margins command in Stata.

As a first approximation, marginal log-hazards can be turned in to marginal hazards by exponentiating the margins.

**Marginal log-hazard for counterfactual ACEs intervention by ACE count group**

If, in the previous section, only those participants who had a specific number of ACEs had been selected, the same logic applies.

**Constant of proportionality**

Those who have an ACE count of zero have no change to their ACE variables made by the counterfactual intervention. They are a reference group, and the marginal log-hazards of the intervention is zero for them. For groups with higher number of ACEs, we want to estimate how their marginal log-hazard increases in proportion to the number of ACEs.

Let $z_{k}$ be the average covariates for the subgroup of participants with k ACEs, and $z_{k}'$ be the average intervened upon covariates (as described above). Then, for the kth group, the marginal log-hazard is

$$\beta^{T}(z_{k}^{'} -z_{k})$$

The estimate for the constant of proportionality between the above expression and k is given by

$$\frac{\sum_{k} k\beta^{T}(z_{k}^{'} -z_{k})}{\sum_{k} k^{2}}=\beta^{T}\frac{\sum_{k} k(z_{k}^{'} -z_{k})}{\sum_{k} k^{2}}$$

It is a linear combination of the coefficients $\beta$, and therefore using $\beta$’s estimate and covariance we estimated the constant of proportionality between the number of ACEs and the marginal log-hazards for the related subgroup.

Again, the only variables which contribute to this calculation are the ACE variables, and their counterfactual values, as an average.
